# *Vibrot*, a Simple Device for the Conversion of Vibration into Rotation Mediated by Friction: Preliminary Evaluation

**DOI:** 10.1371/journal.pone.0067838

**Published:** 2013-08-05

**Authors:** Ernesto Altshuler, Jose Martin Pastor, Angel Garcimartín, Iker Zuriguel, Diego Maza

**Affiliations:** 1 “Henri Poincarè” Group of Complex Systems, Physics Faculty, University of Havana, Havana, Cuba; 2 Departamento de Física y Matemática Aplicada, Facultad de Ciencias, Universidad de Navarra, Pamplona, Spain; University of Zurich, Switzerland

## Abstract

While “vibrational noise” induced by rotating components of machinery is a common problem constantly faced by engineers, the controlled conversion of translational into rotational motion or vice-versa is a desirable goal in many scenarios ranging from internal combustion engines to ultrasonic motors. In this work, we describe the underlying physics after isolating a single degree of freedom, focusing on devices that convert a vibration along the vertical axis into a rotation around this axis. A typical *Vibrot* (as we label these devices) consists of a rigid body with three or more cantilevered elastic legs attached to its bottom at an angle. We show that these legs are capable of transforming vibration into rotation by a “ratchet effect”, which is caused by the anisotropic stick-slip-flight motion of the leg tips against the ground. Drawing an analogy with the Froude number used to classify the locomotion dynamics of legged animals, we discuss the walking regime of these robots. We are able to control the rotation frequency of the *Vibrot* by manipulating the shaking amplitude, frequency or waveform. Furthermore, we have been able to excite *Vibrots* with acoustic waves, which allows speculating about the possibility of reducing the size of the devices so they can perform tasks into the human body, excited by ultrasound waves from the outside.

## Introduction

Undesired vibrations associated to rotational parts in machines are widely studied in engineering, but the studies of the inverse effect (i.e. vibration induced rotation) are limited. Amateur videos demonstrating this strategy can be found in the web [1] which display funny robots performing an erratic walk when shaken. Despite the apparent simplicity of these devices, the physical mechanism behind their dynamics is subtle and complex [2]. In 2001 a practical way to reduce the time of fastener-insertion in industrial processes was proposed [3] and in the same year, a gadget converting longitudinal oscillating or fluctuating motion into a unidirectional rotation was constructed and characterized theoretically in detail [4], [5]. More recently, conversion of piezoelectric vibration into rotation by friction has allowed the design of a new generation of motors (see, for example, [6]). A number of “jumping robots” that transform rotational energy of an inner part into translational energy have aimed, for example, at the exploration of very low gravity asteroids [7]. In this paper, we propose a very simple “robot” (called *Vibrot*) able to convert a vertical vibration relative to the ground, into rotational motion around an axis perpendicular to the ground. In a *Vibrot*, the conversion from vibrating to rotating energy is mediated by an asymmetric friction against the ground, and by elastic energy temporarily stored in the legs–which resembles the biomechanics of running [8]–[10]. We experimentally quantify the main features of the *Vibrot*, and propose an alternative mechanism to the conversion of vibrational into rotational motion with important practical applications.

## Materials and Methods

**Figure 1:**
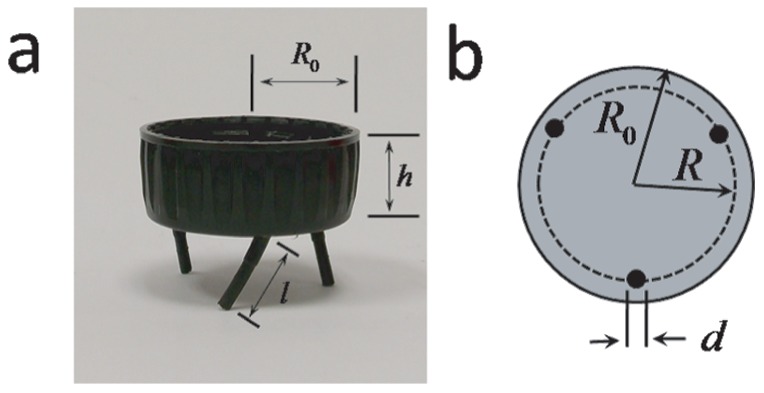
A typical *Vibrot*. (a) Photograph and labels of the relevant dimensions. (b) Sketch of the bottom view.

A typical *Vibrot* is presented in Fig. 1. It is a remarkably simple device that can be built from a soft drink bottle cap. The external radius of the cap is 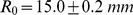 and its height is 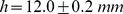 (Fig. 1.a). Three rubber legs were glued with cyanoacrylate on the flat side of the cap, at three points forming angles of 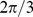 relative to the symmetry axis of the cylinder, each located at a distance 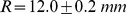 from the center (Fig. 1.b). The legs made angles of 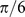 relative of the normal of the bottom wall, in a ratchet configuration (Fig. 1.a). Each leg is a neoprene-rubber cylinder of length 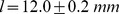, a diameter 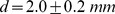 and mass 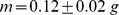. The cantilever spring constant of one leg was measured to be 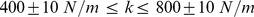 (the lower and upper limits correspond to forces exerted at the tip of the leg to the left and to the right, taking as a reference the foremost leg in Fig. 1(a)). The total mass of a *Vibrot* could be changed by adding concentrically metal washers inside the cap. Other working *Vibrots* different from the ones described here can be constructed using a wide range of materials and geometries. In addition, by changing the legs symmetry, a *Vibrot* can be converted into a “runner”, but those are beyond the scope of this paper.

For characterization, *Vibrots* where set on a horizontal, circular acrylic platform firmly attached to a TIRA TV52120 vibrator, fed by an amplified Arbitrary Waveform Generator (Agilent 33220A). The vertical acceleration of the platform was measured using an accelerometer conditioned by a PCB Piezotronics model 482C, which output was read using a digital oscilloscope. So, both the vibration frequency of the platform 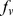, and its maximal dimensionless acceleration 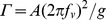 (where 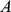 and 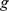 are the vibration amplitude and the gravitational acceleration, respectively) were fully controlled. The data reported in this work has been collected for sinusoidal vibrations, but *Vibrots* can also rotate when driven by square, sawtooth and non-periodical waveforms. *Vibrots* were filmed laterally using a high speed camera Photron Fastcam 1024PCI model 100 K, within a speed range from 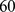 to 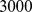 frames per second (fps).

## Results and Discussion

In a typical experiment, a *Vibrot* was left standing on the vibrating plate at given values of 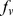 and 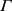 (video S1 and S2). Then it started to rotate around its vertical symmetry axis at a fairly constant frequency 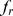, which was measured by means of spatial-temporal diagrams (these are built by stacking a horizontal line of pixels from the frames of a video shot from the side).

**Figure 2:**
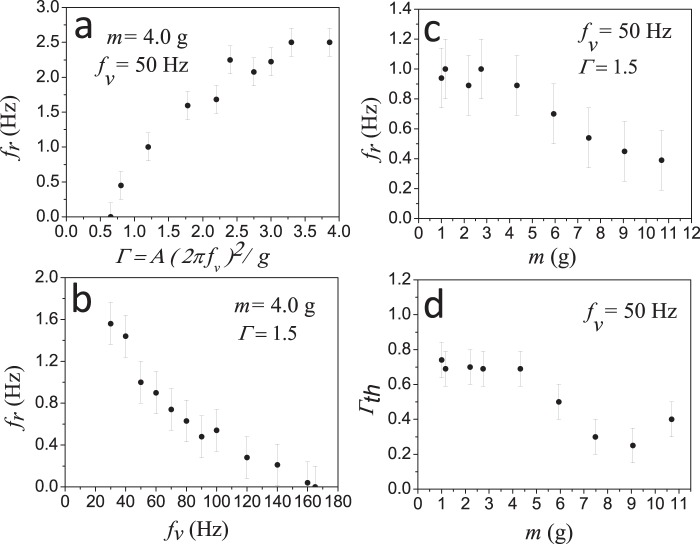
Basic characterization of the *Vibrot* motion. (a) Dependence of the rotation frequency on the vibrating plate's adimensional acceleration, for a fixed frequency excitation. (b) Dependence of the rotation frequency on the vibration frequency, at a fixed adimensional acceleration. (c) Rotation frequency as a function of the *Vibrot* mass for constant vibration frequency and adimensional acceleration of the platform. (d) Threshold adimensional acceleration as a function of the *Vibrot* mass, at a fixed vibration frequency. (In this plot, the point corresponding to the biggest mass is associated to a high deformation of the legs).

Let us start by characterizing the dependence of the *Vibrot* kinematics on the main control parameter: the dimensionless acceleration of the platform 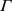 (Fig. 2.a). As could be intuitively expected, the rotation frequency increases as the shaking amplitude is increased, with the vibration frequency fixed at 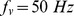. From this figure, three important features can be distinguished: (i) there is a threshold value for 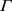 below which the *Vibrot* is not “activated”; (ii) just above the threshold 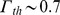, the rotation frequency increases with 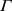 with no further changes until (iii) saturation, at 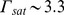. These three features remain essentially the same for slight geometrical variations of the *Vibrot*.

Fig. 2.b indicates that for a fixed value of 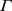 the angular velocity of the *Vibrot* decays as the vibration frequency increases. Let us consider a simple argument in order to understand qualitatively this result. From a macroscopic point of view, the vibrating platform injects in the *Vibrot* an amount of energy which must be proportional to the maximum kinetic energy delivered by the shaker 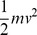 (where 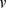 is the maximum shaker velocity). This energy, that can be expressed more conveniently as 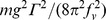, is converted into elastic energy by the legs, which finally drives the displacement of the *Vibrot* in the azimuthal direction. Assuming that the vertical and rotational energy are proportional, we obtain 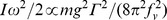 (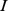 is the moment of inertia of the *Vibrot*). Therefore 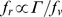, which is reasonably consistent with the results displayed in Fig. 2.a. and Fig. 2.b.

**Figure 3:**
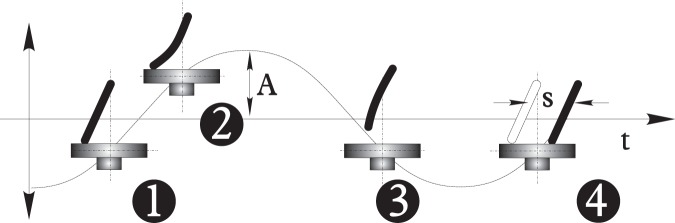
Qualitative stride mechanism. Sketch of the motion of one *Vibrot* leg near the vibrating platform. The solid line represents the trajectory of the vibrating plate (*grey*) during one cycle (Video S3).

Figure 2.c shows that the frequency of rotation depends on the mass of the *Vibrot* except for very low masses. Direct inspection of videos taken at 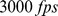 allowed us to explain this behavior, by getting insight into the rotation mechanism based on the motion of each leg. In what follows we describe this mechanism qualitatively during one vibration cycle. As the platform rises, the leg bends laterally as a cantilever, so elastic energy is stored in it (Fig. 3, stages 1 and 2). Then the *Vibrot* detaches from the base flying freely in the vertical direction while the leg releases its elastic energy and advances to the right (video S3). If the mass is small, the flight takes comparatively more time than sliding. As the mass is increased the difference is reduced, and this may explain the decrease of 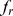 with mass, as displayed in Figure 2.c. Finally, the leg lands on the platform to the right of the starting point (see Fig. 3, stage 4); we will call a *stride* the distance 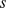 between the two points. It is worth noticing that a device called *Bristle*–*bot* (aimed at translation instead of rotation) is based on elastic legs whose working principle is roughly the same as our *Vibrot* legs, and has been worked out theoretically in [2] very recently.

We underline, however, that the precise stride mechanism is nontrivial and will be revisited in future works. Still, our simplified description strongly resembles animal running [9], and perhaps more evidently, human running with the use of lower-limb prosthetics [10]. Assuming the analogy with a walking creature, we can calculate the Froude number 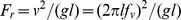 for our “animal”, where 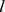 works as a characteristic length equivalent to the distance between foot and hip in actual runners. For the rotation frequencies observed in this work (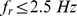) it follows that 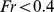. It has been shown [8] that mammals change from walking to trotting at 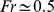, and from trotting to galloping at values higher than 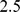. Nevertheless, a careful inspection of the high speed movies evidences that, regardless of 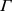, our *Vibrots* are “galloping” (with all the legs detached from the floor during part of the cycle, except for very high masses). This fact is a clear indication that 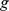 is not the correct scale for the potential energy involved in the walking mechanism. Instead, leg deformation probably determines this scale, although its quantification is not an easy task.

Figure 2.d displays a rather puzzling behavior, namely that the acceleration threshold needed to observe rotational motions *decreases* as 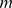 is increased. Nevertheless, a reasonable explanation can be given: a large enough “lateral push” from the ground is required to start the rotational motion, and this push is provided by the static friction force, which increases with mass. Let us remark that the point corresponding to the highest mass in Fig. 2.d cannot be entirely trusted, since the video showed for that case that the legs changed sharply their geometry due to the large load.

We will now analyze the dynamics displayed by the *Vibrot* in a single step. The distance covered by one point of the *Vibrot* perimeter during a single vibration cycle is the stride 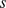, as defined before. Hence, a point on the perimeter will move tangentially at a speed 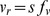. Besides, the kinematics of rotation imposes 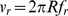. Combining both expressions we get:
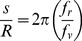
(1)


**Figure 4:**
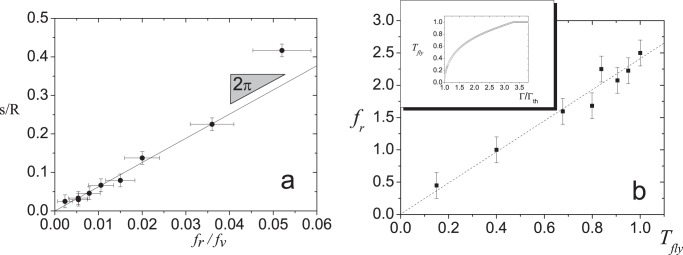
Dependence of the stride length on the flying time. (a) Stride length dependence on the frequency ratio, 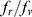. The slope of the solid line is 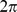. (b) Frequency of rotation as a function of the flying time, 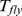 (as a fraction of the period 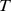). The doted line is just a guide to the eye. In the inset, the flying time (as a fraction of the period 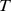) is shown versus the acceleration for an inelastic bouncing object. See text for details.

Notice that the above formula is based on a purposely simple model, where slip of the legs on the surface, as well as their possible back-forth and radial motions associated to a potentially nonlinear elastic response and construction defects are ruled out. All these factors could make that the magnitude of the stride length were erratic which would imply that 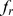 is not stable. Nevertheless, Fig. 4.a shows that the *average* stride length (extracted from high speed recordings) versus the ratio of rotational to vibrational frequencies is nicely fitted by a straight line with a slope of 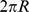. This is a clear indication that Eq. 1 is a good description of the *Vibrot* kinematics, and evidences that the stride 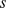 is the relevant variable of it for most of the frequency range under study. However, figure 4.a suggests that Eq. 1 departs from the experiment at high (and probably also low) 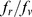 values.

Finally, as 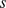 is governed by time the *vibrot* is flying, 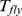, the time elapsed since it detaches from the vibrating base until it lands. The flying time of a bouncing particle on a shaking base has been introduced as paradigmatic example of complexity on non–linear dynamics [11]. Let us check the dependence of the *Vibrot* tangential velocity on this time. For the sake of simplicity, let us assume that the flying time of the *Vibrot* has the same dependency on 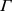 as the one obtained for the completely inelastic case [12]. 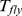 can be numerically calculated as a function of 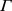 (see inset in Fig. 4.b). The flying time has been obtained for the values of 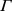 explored in 2.a, and 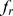 has been plotted as a function of 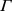 in Fig. 4.b. The relationship obtained indicates that the angular velocity depends linearly on the *Vibrot* flying time.

## Conclusion

The results and the simple arguments introduced in this paper indicate that the mechanism responsible for the rotational movement of the *Vibrot* is related to the asymmetry introduced by the static friction between the legs and the base. In fact, in order to make the *Vibrot* rotate, it is essential that the legs bent to one side when attached to the vibrating plate. This deformation stores elastic energy. Then, after detaching from the base, the energy is released and the *Vibrot* can freely rotate while flying. In order to validate this idea and to explore the capability of these devices as practical motors, we devised a different vibration source, building *Vibrots* with a horizontal membrane attached on top, which acts as an “antenna” for acoustic waves. This has proven to provide enough vibrational energy to make the device rotate on a non-vibrating horizontal surface. Importantly, *Vibrots* with different sized antennas rotate only when they resonate at certain frequencies or frequency ranges (video S4).

In conclusion, we have built and experimentally characterized a device able to rotate by extracting energy from vibration. The frequency of rotation can be controlled by tuning the frequency and/or adimensional acceleration of the vibration. While the details of the *Vibrot* motion will be the subject of future work, we have already found similarities between the *Vibrot* legs and those of running mammals, which are very promising in the field of biomechanics. Moreover, if an appropriate technique is set up to miniaturize *Vibrots*, their working principle could be used, in principle, to fabricate ultrasound-propelled micro-drills for medical or other practical uses.

## Supporting Information

Video S1 **Experimental set–up description and explanation of the movement of the** ***Vibrot*** **induced by vibration.**
(MPG)

Video S2 **Typical rotation movement displayed by** ***Vibrot*** **when is subjected to vertical shaking.** The following shows the process through which the space-time diagrams are obtained in order to measure the rotation frequency.
(MP4)

Video S3 **Detailed dynamics of the** ***Vibrot*** **using high speed camera.**
(MP4)

Video S4 ***Vibrots*** **with a horizontal membrane attached on top are excited by acoustic waves on a non vibrating surface.** They are able to rotate only when resonate at certain frequency ranges.
(MP4)
